# Decoding the evolution of melodic and harmonic structure of Western music through the lens of network science

**DOI:** 10.1038/s41598-026-42872-7

**Published:** 2026-04-23

**Authors:** Niccolò Di Marco, Edoardo Loru, Alessandro Galeazzi, Matteo Cinelli, Walter Quattrociocchi

**Affiliations:** 1 Department of Legal, Social, and Educational Sciences, Tuscia University, Viterbo, Italy; 2 Department of Computer, Control and Management Engineering, Sapienza University of Rome, Rome, Italy; 3 Department of Mathematics, University of Padova, Padova, Italy; 4 Department of Computer Science, Sapienza University of Rome, Rome, Italy

**Keywords:** Computational science, Computer science, Applied physics, Statistical physics, thermodynamics and nonlinear dynamics, Evolution

## Abstract

Music has always been central to human culture, reflecting and shaping traditions, emotions, and societal changes. Therefore, analysing the quantitative properties of musical compositions can provide insights into specific aspects of human cultural evolution. In this study, we conduct a large-scale analysis of approximately 20,000 musical pieces rooted in Western musical practices. The resulting dataset encompasses MIDI transcriptions of works from six major macro-genres, spanning nearly four centuries of musical history. We model musical composition as weighted directed networks, enabling a systematic investigation of melodic and harmonic properties through a network-based representation of music. Our results show that different genres have distinct topological and musical properties, providing valuable insights into the origins of their differences. Moreover, a temporal analysis reveals systematic changes in network-based measures, suggesting a trend toward increasing similarity and reduced complexity in the melodic and harmonic structures. Notably, within this analytical perspective, even long-established and structurally complex genres such as Classical and Jazz display patterns that are increasingly comparable to those of more recent genres.

## Introduction

Music is a defining element of human culture, reflecting and shaping traditions, emotions, and societal changes^1–3^. Its capacity to engage cognition and emotion has long intrigued researchers across disciplines^4–10^. Over centuries, it has evolved alongside cultural and technological shifts, adapting to new tools and contexts of creation and consumption^11^.

Historically, music was a communal experience, limited to live performances and closely tied to specific cultural practices^12^. Moreover, composing music was restricted mainly to trained specialists, often working within traditional frameworks. The invention of new technologies revolutionized this dynamic, allowing music to transcend temporal and spatial boundaries^13^. By the mid-20th century, physical formats like vinyl records and cassette tapes had democratized access to music and enabled a broader audience to participate in music creation, laying the groundwork for today’s digital era^14–16^.

This shift has fostered the emergence of new genres and innovative styles, challenging traditional notions of musical expertise and redefining creative boundaries^17^. Moreover, the recent advent of streaming platforms and social networks has reshaped our cultural landscape^18,19^, including how music is consumed and produced^20^.

Platforms like Spotify and YouTube not only offer listeners personalized recommendations but also act as hubs for discovering and promoting new artists, effectively functioning as the “new radio” of the digital age^21–23^. Algorithms play a pivotal role in shaping these experiences, tailoring music discovery to individual preferences and thereby influencing listening habits^24–27^.

However, this interconnected landscape is not without its drawbacks. Previous studies have suggested that content circulating in fast, interconnected, and algorithmically curated environments is subject to simplification processes, as seen in the case of song lyrics^28^ and social media comments^29^. This raises an important question: is a similar trend occurring in the musical landscape?

Musicians and musicologists have highlighted a general trend of musical simplification from a theoretical perspective^30–32^. However, addressing this question within a scientific, data-driven framework is challenging due to the lack of standard methods for measuring musical complexity^33–35^.

Despite these challenges, the emergence of large collections of musical scores, recordings, and lyrics has kickstarted a period of prolific musical and art research^17,36–40^. Notably, multiple studies within this growing body of work attempt to analyze musical evolution. For example, the authors of^41^ studied how pitch, timbre, and loudness evolve in the last 50 years, finding a reduction in pitch variety, a timbre homogenization, and an increase in the loudness of songs. Similar results were obtained in^42^, where the authors find a decreasing complexity and increasing note density in popular melodies over time. Conversely, focusing on popular music in the USA, the authors of^43^ found no evidence of progressive homogenization, suggesting instead that popular music changes in response to specific human events. Furthermore, special attention has been paid to study how the inharmonicity and noisiness have evolved in contemporary music^44^, finding that recent popular music is both more inharmonic than Western Classical music and experimental music, such as *musique concréte*. However, much of this literature focuses on relatively recent repertoires or relies on limited datasets, leaving long-term structural patterns across broader historical periods less explored.

**Fig. 1:**
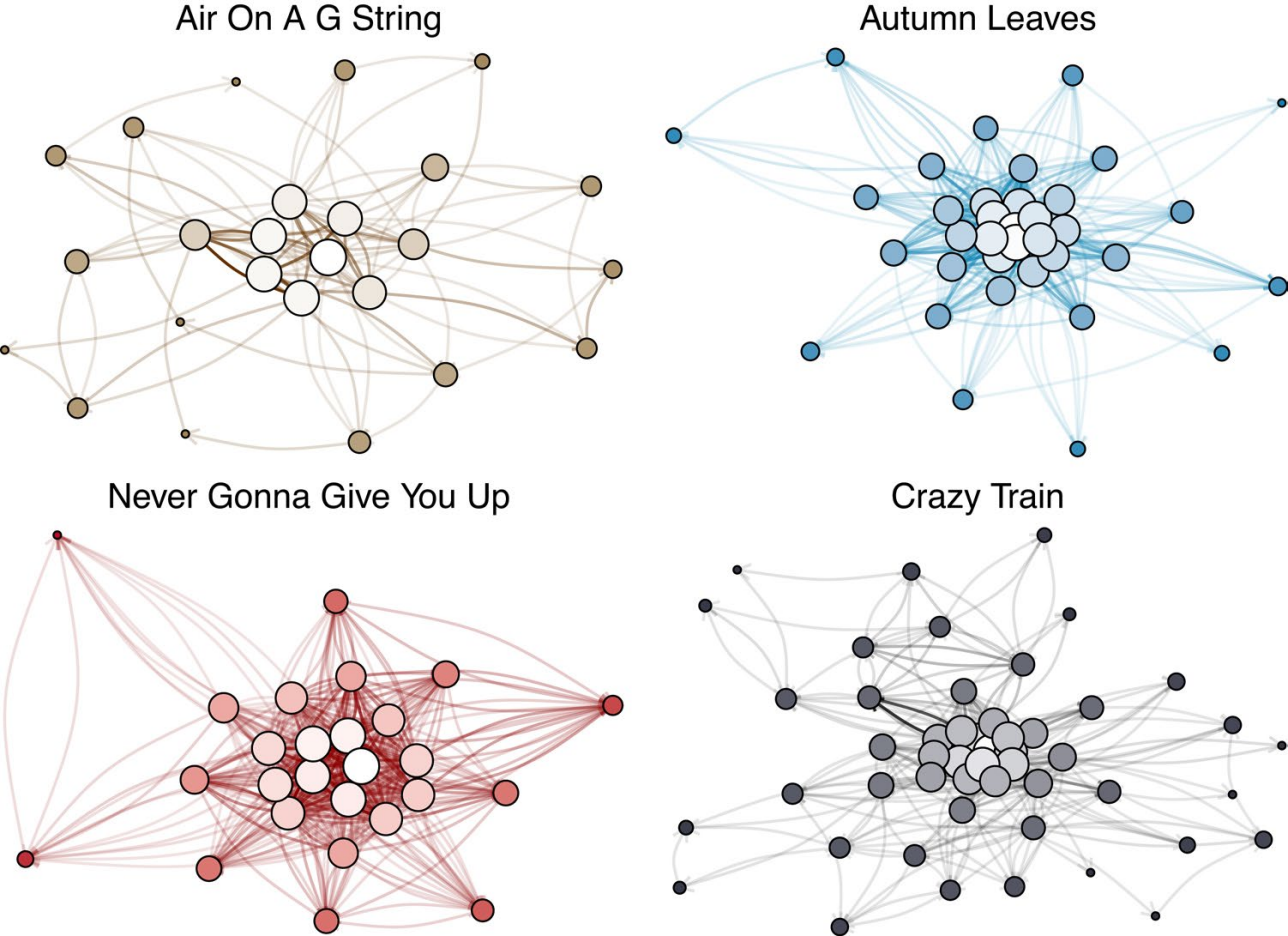
Networks constructed from four MIDI files. The size and the brightness of each node are proportional to the degree. The transparency of each edge is inversely proportional to its weight.

To fill this gap, in this work we build upon the approach of previous studies^45–47^, utilizing Network Science tools to analyze musical compositions. In particular, we analyze a dataset of approximately 20, 000 MIDI of pieces rooted in Western musical practices, categorized into six macro-genres^48^ and spanning four centuries of composition. In particular, we represent musical compositions as weighted directed networks where notes are nodes and transitions are edges, as depicted in Fig. 1.

This approach is not without drawbacks. MIDI files come with inherent limitations, such as their reliance on a discrete twelve-tone pitch system^49–52^, which in turn makes it difficult to transcribe music strongly relying on sound design over note selection accurately^49,53^. Therefore, our analysis focuses on melodic and harmonic transitions, deliberately setting aside other dimensions of musical complexity that are not measurable by our approach.

Nevertheless, our methodology offers a way to explore structural differences across Western music tradition, providing a framework for measuring musical complexity and its evolution over time.

Our analysis reveals that Classical and Jazz compositions obtain higher values of complexity, indicating greater structural diversity in melodic and harmonic transitions compared to other genres. Furthermore, unlike previous studies, our measures offer a comparison of the topological and musical features defining each genre, shedding light on the foundational elements of their identities. Finally, we conduct a temporal analysis to examine whether systematic changes have occurred over time. Notably, we find that long-established and traditionally more complex genres such as Classical and Jazz exhibit structural patterns that increasingly resemble those of more recently developed genres, suggesting a process of homogenization and simplification in melodic and harmonic structures.

Rather than providing direct evidence for specific causal mechanisms, our findings are consistent with hypotheses that recent technological advancements, progressive democratization, and the development of highly interconnected environments may be associated with changes in certain structural aspects of music, in a manner analogous to patterns observed in other cultural domains^28,29^. At the same time, these developments may have encouraged musical practices to explore new expressive spaces and alternative forms of complexity that are not captured by the present framework. We emphasize that these interpretations are speculative and contextual: the present study does not directly test them, and the observed patterns may result from multiple interacting factors, including those highlighted in prior work^43,54–57^. Finally, the trends discussed here pertain specifically to symbolic, pitch-based representations of music and may not generalize to musical traditions that do not rely on the twelve-tone pitch system, nor to genres in which salient musical features lie outside the dimensions considered in this study. Future work could integrate multiple complementary approaches—combining symbolic, audio-based, and contextual representations—to examine how different dimensions of musical complexity interact over time and to assess whether broader, multidimensional notions of musical complexity exhibit coherent evolutionary patterns.

## Results

### Genre-specific network properties

Before the main analysis, we recall that our dataset contains $\approx 20.000$ networks in which notes are nodes and edges are transitions among them.

**Fig. 2:**
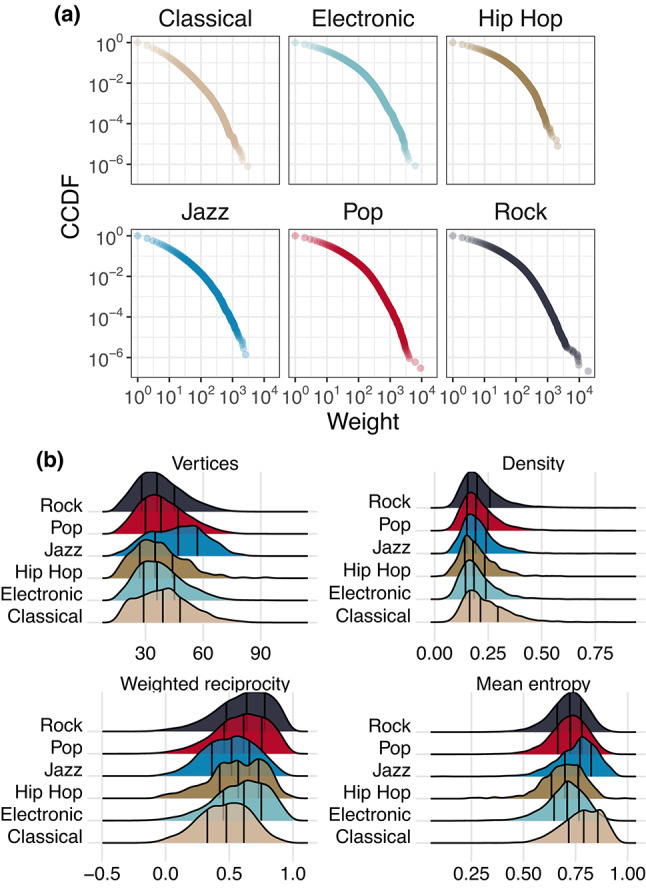
(**a**) CCDF of aggregate network’s weights. (**b**) Distribution of various measures, divided according to the genre. The vertical bars represent the quartiles of each distribution.

The first natural question is whether networks from different genres exhibit distinct properties. To explore this, Fig. 2 presents several measures calculated on our collection.

Figure 2a shows the CCDF of all the edge weights appearing in each genre. Notably, all distributions are heavy-tailed, but we observe that Electronic, Pop, and Rock genres exhibit longer tails than Classical and Jazz.

Figure 2b illustrates the distributions of several network metrics grouped by music genre, namely the number of vertices, density, weighted reciprocity, and mean node entropy. See Methods for each metric’s definition and their musical interpretation.

Regarding vertex count, Jazz pieces stand out with a higher number of notes, while other genres consistently exhibit fewer notes. Interestingly, all genres share similar density values. This property may be tied to musical patterns that lead a note to be connected only to a limited number of other notes.

For weighted reciprocity, more pronounced differences emerge: Rock, Pop, Hip Hop, and Electronic music exhibit very high reciprocity, whereas Classical and Jazz show lower median values and, in some cases, even anti-reciprocity. This pattern is consistent with Classical and Jazz pieces exhibiting less repetitive bidirectional note transitions, whereas other genres display more frequently recurring transition pairs.

Finally, the higher entropy values observed in Jazz and Classical imply nearly uniform transitions between notes. This indicates that, on average, the transitions between a source node and its targets occur with similar frequency. Interestingly, this points to the fact that musical connections are pre-established among harmonious notes and are likely to occur fairly uniformly.

To test the previous insights formally, we run two-sample Mann–Whitney U tests^58^ between each pair of genres, adjusting the *p*-value using the standard Bonferroni-Holm correction^59^. The results, shown in Figure S1 in Supplementary Information (SI), are aligned with our insights.

### Analyzing musical connectivity and variability

**Fig. 3:**
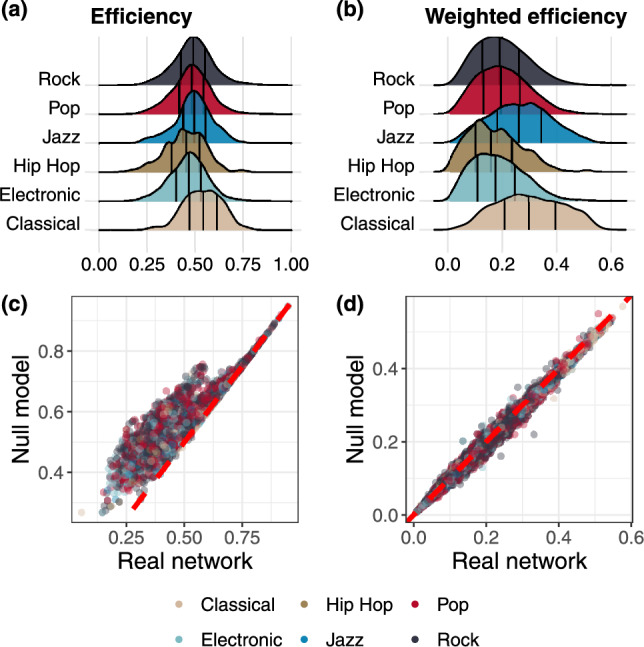
(**a**) Distribution of unweighted efficiency. (**b**) Distribution of weighted efficiency. (**c**), (**d**) Comparison between efficiency and weighted efficiency in real and randomized networks. To improve their visualization we use a random sample of $n = 10^4$ points.

In this section, we focus on the spreading properties of networks using the measures of *global efficiency* (see “Methods” section for further details). Figure 3a,b shows the distributions of efficiency and its weighted counterpart for each genre. Recall that these values are connected to the shortest paths of the network and, particularly in the weighted case, capture structural properties related to the organization of note-to-note transitions. As detailed in the “Methods” section, high weighted efficiency values are associated with musical pieces exhibiting greater variability in note sequences, thus measuring our intended measure of complexity. Figure 7 illustrates two networks with different efficiency values, providing a visual representation of how this measure reflects differences in melodic and harmonic organization encoded in the network structure.

Hip Hop and Classical music stand out among other genres regarding non-weighted efficiency. Specifically, Hip Hop is characterized by lower efficiency values, whereas Classical music typically exhibits higher values. These differences become even more evident when weights are considered, i.e. the number of times a transition between two notes occurs. Notably, we observe the greater efficiency of Classical and Jazz structures and lower values of Hip Hop.

Given that all weights satisfy $w_{ij} \ge 1$, higher efficiency corresponds to shortest paths carrying low weights. Since weights represent the number of times a transitions occour between notes, this indicates less concentration on a small subset of highly repeated transitions and a more even exploration of available note sequences. Within this framework, higher weighted efficiency values observed for Classical and Jazz reflect greater structural diversity in melodic and harmonic transitions, as compared to other genres.

Interestingly, the similarities observed in non-weighted efficiency suggest that while the average distance between two notes is comparable across all genres, their differences lie in the weighted structure of the networks.

To deepen our exploration, we compare the observed efficiency with that derived from ad-hoc null models (see Methods for further details). Briefly, the first null model rewires the edges between notes, preserving the out-degree distribution. The second one shuffles the weights among each node’s out-edges, maintaining the out-strength distribution.

The results of this analysis are depicted in Fig. 3c,d.

Panel (c) reveals that, in nearly all cases, the efficiency values in real networks are lower than those obtained from a random topology where edges are rewired but the number of transitions from each note is preserved. Conversely, panel (d) displays values comparable to a null model that preserves the out-strength of each node.

These results highlight some interesting musical patterns. In fact, the edge rewiring procedure allows transitions that may be dissonant, breaking common musical patterns. At the same time, it creates shorter paths, resulting in greater efficiency. Interestingly, this observation suggests that musicality does not necessarily prioritize the formation of short paths. On the other hand, rewiring only the weights maintains the overall musical structure, changing only the number of transitions between notes. However, the comparable results with a null model may be a consequence of the high values of entropy (shown in Fig. 2b).

To check the robustness of our results, Figure S2 contains the results of the same analysis using a different measure, the *Network Entropy*, previously used in other work to quantify the information contained in musical networks constructed from J.S. Bach pieces^45^. Moreover, we also considered an alternative measure, called *Effective Resistance*, whose results are shown in Figure S4. Notably, for both measures, we observe similar patterns, with Classical and Jazz music obtaining higher complexity values than other genres. For a full discussion on the measure and the results, see Section “Analysis with alternative measures of complexity” in SI.

### Embedding musical structures in high-dimensional spaces

Up to this point, our analysis has primarily examined musical networks from a purely topological perspective, followed by an interpretation of the results from a musical standpoint. However, this approach may not be sufficient to fully capture the networks’ properties. To this end, we also incorporated musical information contained in musical intervals.

Recall that an interval corresponds to the difference in pitch between two notes, measured as the number of semitones between the two. In Western music theory, the main intervals are 12 and range from “perfect unison” (0 semitones of difference) to “major seventh” (11 semitones of difference). Table 1 provides a complete list of these intervals and their corresponding differences in semitones.

**Table 1:** Musical intervals and their distances in semitones.

Interval	Semitones
Perfect Unison	0
Minor Second	1
Major Second	2
Minor Third	3
Major Third	4
Perfect Fourth	5
Tritone	6
Perfect Fifth	7
Minor Sixth	8
Major Sixth	9
Minor Seventh	10
Major Seventh	11

**Fig. 4:**
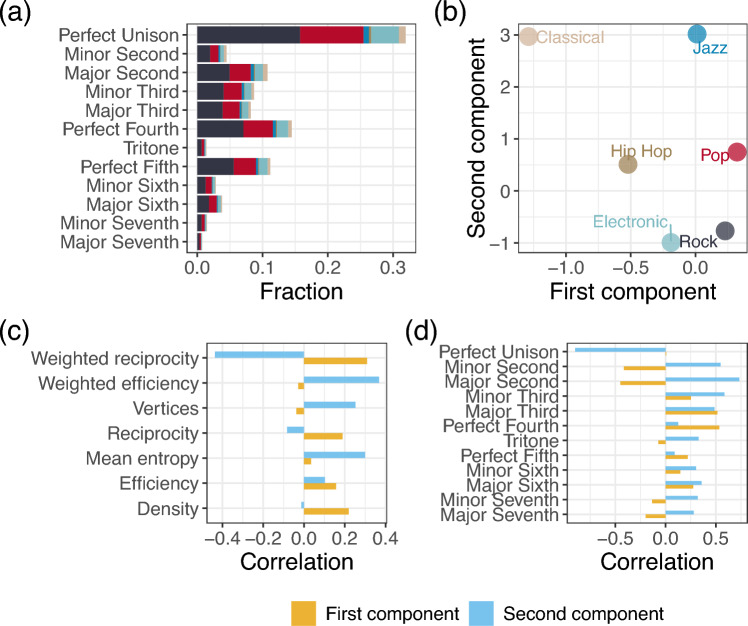
(**a**) Fraction of intervals appearing in each musical genre. (**b**) Center of mass for each genre, computed using UMAP 2-dimensional coordinates. (**c**) Correlation between components and measures. (**d**) Correlation between intervals entries and components.

How frequently these intervals occur within a musical piece reflects its musical properties, such as its key and the mood it may evoke in a listener. To give an idea of intervals appearing in our dataset, Fig. 4a shows the fraction of intervals appearing in pieces, divided by genres. Notably, our results are coherent with those obtained in previous work^11^.

In the Sec. “Network embeddings” of methods, we explain in detail how we construct interval embeddings of our dataset. Briefly, we associate to each *G* a vector $v_G$ whose components are linked to the number of times a specific interval appears in the musical piece, not distinguishing among notes at different octaves.

To check the robustness of these results, in SI we repeat the analyses of this section using graph2vec, an algorithm that embeds networks in a high-dimensional space using their topological properties. The results, shown in Figures S8 and S9 of SI, exhibit consistent outcomes, demonstrating that our findings are robust and can be achieved using either topological or musical properties. As a further analysis, Figure S10 in SI also compares the embeddings of real networks with the ones created from randomized versions of networks, observing clear differences between the two. These results corroborate the efficacy of our procedure in capturing relevant musical properties of networks.

Figure 4b shows the center of mass of each genre, computed starting from the 2-dimensional representation of UMAP^60^. We report the plot containing all the points in Supplementary Fig. S7, where, although most genres appear mixed up, a clear cluster for Classical music emerges, highlighting its difference from other genres.

These differences become even clearer in Fig. 4b, revealing a notable distance between Jazz and Classical from other genres.

To understand what network properties each component embeds, in panel Fig. 4c we show the Pearson correlations between the coordinates of musical pieces on the UMAP plane and their measured network properties. The first component shows significant correlations only with topological properties such as efficiency ($r \approx 0.16, p < 0.001$), density ($r \approx 0.22, p < 0.001$), and reciprocity ($r \approx 0.18, p < 0.001$). On the other hand, the second component is correlated mostly with weighted properties such as weighted efficiency ($r \approx 0.37, p < 0.001$), mean entropy ($r \approx 0.30, p < 0.001$), and weighted reciprocity ($r \approx -0.44, p < 0.001$). While the first component appears to reflect a combination of topological properties, the second one captures the weighted structure and complexity of the musical pieces. These findings align with the results presented in Fig. 2. Moreover, while Classical and Jazz share similar values on the second component (i.e. similar complexity), they differ on the first, suggesting that their differences may be rooted in topological properties.

Conversely, to assess which intervals contribute to the two components, we compute the correlations between the coordinates and each component of the $12-$dimensional vector. Mathematically speaking, let us consider the $N \times 12$ matrix $M = [\mathbf{v_j}]_{j = 1\ldots 12}$, where *N* is the number of songs and each row represents the vector associated with a composition. We compute the correlations $r(\textbf{v}_j, pr_i), j = 1 \ldots 12, i = 1,2$ where $pr_i$, denotes the coordinates of $i-$th component according to UMAP.

The resulting values are reported in Fig. 4d. We find that the first component is strongly influenced by the presence of major thirds and perfect fourths, while the second component is predominantly associated with minor and major seconds, as well as unisons. These relationships help identify which intervals are more characteristic of specific genres.

For instance, both Classical and Jazz compositions frequently rely on unisons and minor/major seconds and thirds, but they differ in their emphasis on major thirds and perfect fourths. Similarly, genres like Hip Hop-Pop, Electronic, and Rock exhibit comparable patterns in their interval distributions, though with distinct nuances.

A further interpretation of the two dimensions comes from Western music theory, where intervals are commonly classified as either “stable’ or “unstable’ based on their tendency to resolve. Resolution refers to moving from a state of dissonance that might evoke incompleteness or suspense to one of consonance that feels definitive and accomplished. Stable intervals, such as the unison and perfect fifth, are consonant and do not require resolution, providing a sense of repose. Imperfect consonances like major and minor thirds and sixths are also stable but slightly less complete. In contrast, unstable intervals, including major and minor seconds, major and minor sevenths, and the tritone, create tension and seek resolution to more stable intervals, such as thirds or octaves. With these notions in mind, we observe that the first component is negatively correlated with unstable intervals and positively correlated with stable ones. Conversely, the second component exhibits mostly stronger correlations with unstable intervals and weaker correlations with stable ones.

### Tracing musical evolution over time

In the previous sections, we analyzed the networks’ properties without considering the release period of each musical piece. Here, we incorporate this additional dimension to explore the temporal evolution of musical pieces.

**Fig. 5:**
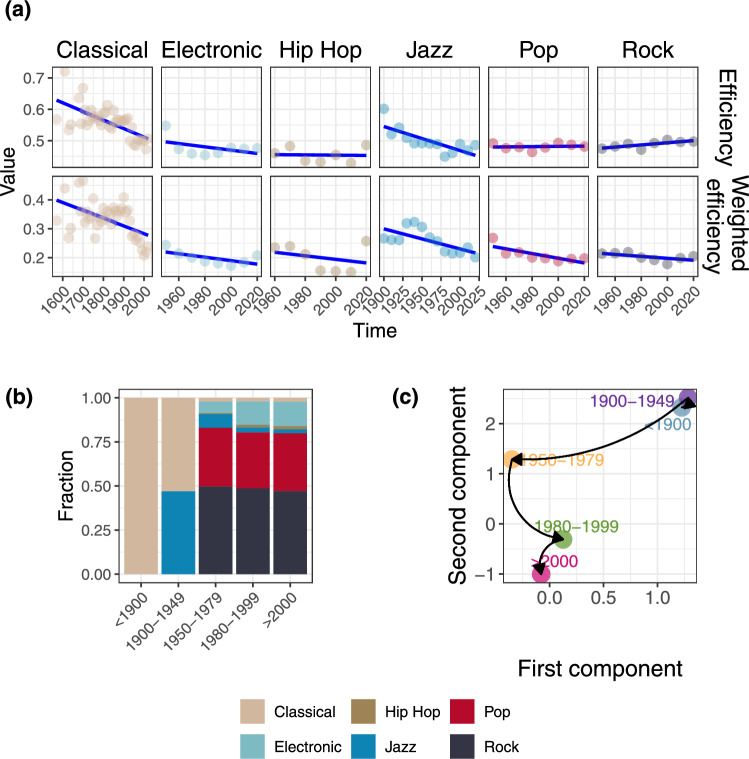
(**a**) Evolution of mean efficiency measures over decades. The arrows highlight the temporal evolution of considered eras. (**b**) Distribution of genres in each musical era. (**c**) Center of mass of each musical period, obtained using UMAP on the interval embeddings.

Since the Spotify API often provides incorrect release dates—due to associations with remastered or reissued albums—we developed a heuristic approach using the LLM Gemini to approximate the release date of each piece (details in the Sect. “Release date collection” section of Methods). Our method assigned release dates to 15, 243 pieces, covering approximately $72\%$ of the dataset. Key findings are presented in Fig. 5.

In particular, panel (a) displays the decade-averaged efficiency values for each genre. Notably, Classical music exhibits a declining trend, whereas Jazz shows an initial increase in its complexity in its early days, followed by a decline and eventual stabilization. In contrast, the other genres maintain relatively flat patterns, with efficiency values comparable to those of Classical and Jazz in recent years. To formally validate these observations, Table S2 in SI presents the results of Mann-Kendall tests applied to each trend.

This result suggests a decrease in the complexity of Classical and Jazz music, while the other genres maintain (on average) the same complexity, resulting in values comparable with the ones obtained by Jazz and Classical in recent years. Notably, these results also hold considering the *Network Entropy* and *Effective Resistance* metrics, as shown in Figure S3 and Figure S5. A full discussion of the results in these cases is in Section “Analysis with alternative measures of complexity”’ of SI.

To focus on the musical properties of the MIDIs, we employ the interval embeddings introduced in the previous section. Moreover, we associate each musical piece to one between 5 musical periods, namely $<1900, 1900-1949, 1950-1979, 1980-1999, >2000$. Figure 5b shows the genre distribution among those classes. In particular, we observe a transition from Classical and Jazz music to a prevalence of popular genres such as Rock, Pop and Electronic.

Finally, panel (c) shows the 2-dimensional coordinates of the center of mass of each musical era, where coordinates were obtained using UMAP on the embeddings. The arrows indicate the temporal evolution between musical periods, highlighting a trend starting from the up-right part of the plot through the lower-left quadrant.

To interpret the meaning of the two components, Figure S12 in SI provides the correlation between components with measures and interval occurrences, respectively. The components maintain a similar interpretation of Fig. 4. The first component anticorrelates with topological properties, while the second one is correlated with complexity and weighted properties.

Again, the results highlight a greater complexity (and similarity) for music composed before 1950, and the same holds for recent music, even with lower complexity. Notably, we can observe a transition that, starting from higher complexity, tends to simplicity. At the same time, the differences in the first components may suggest a transition also to different topological properties. Finally, it is interesting to note how recent music shares similar properties, with music composed in the 1950–1979 period acting as a bridge between the two eras.

The observed results could partly be attributed to the rise of more homogeneous and less complex genres in recent years. However, when considered alongside the findings presented in panel (a), our analysis indicates that even enduring genres like Classical and Jazz have undergone a noticeable simplification compared to their origins.

We also acknowledge that Figure S6 and Table S1 in Section “Robustness checks for time analysis” of SI contain further analysis checking that the possible temporal bias in our dataset does not affect the results discussed here.

## Discussion

### Limitations

Our study applies network science and data science to offer a fresh perspective on musical analysis. However, as with all large-scale quantitative approaches, our method is subject to several limitations, which we discuss in this section.

First, our reliance on the MetaMidi Dataset^48^ introduces potential inaccuracies in genre classification and MIDI creations. While genre tagging is helpful for broad categorizations, it may oversimplify the diversity within and across genres. For this reason, genre labels in our study should be interpreted as coarse descriptors rather than precise stylistic definitions.

Second, linking MIDI files to Spotify entries required heuristic methods. Although these heuristics were carefully designed, mismatches or omissions could result in occasional inaccuracies in metadata associations. This issue is especially pertinent for older or less popular tracks, where metadata availability tends to be sparse or inconsistent.

Third, estimating release dates posed a significant challenge since Spotify metadata often fails to reliably indicate the original release dates. To address this, we employed a large language model (LLM) to infer release dates. While innovative, this approach introduces an additional layer of uncertainty, as the LLM relies on contextual data that may be occasionally inaccurate or incomplete.

Lastly, our entire analysis relies on MIDI files, which, as discussed in previous sections, provide a digital transcription of music and therefore a simplified symbolic representation. In particular, MIDI encodes music using a discrete twelve-tone pitch organization, which may not accurately capture approaches such as microtonal music or compositions involving continuous pitch trajectories^49–52^. As a result, the scope of our analysis is inherently restricted to musical dimensions that can be represented symbolically, such as note transitions and melodic and harmonic structure. Musical practices in which pitch is not the primary organizing principle, or in which expressive content is conveyed predominantly through timbre, sound design, performance practice, or continuous pitch modulation, are therefore not adequately represented within our framework. More broadly, other dimensions of musical complexity—including lyrics, timbre, production techniques, sound design, and cultural context—lie outside the scope of the present representation. Consequently, while Western music—and thus the repertoire analyzed here—is largely grounded in the twelve-tone system, our results should not be interpreted as a comprehensive account of musical complexity, but rather as an analysis of specific structural aspects accessible within a symbolic, pitch-based framework. Future research could integrate complementary representations, such as audio-based features, textual analysis, or performance-related descriptors, to provide a more holistic and multidimensional perspective on musical evolution.

Despite these limitations, the robustness of our results across multiple network measures (reported in SI), together with their consistency with prior computational studies, supports the view that the data and methods employed here are appropriate for investigating long-term trends in melodic and harmonic structure within Western music.

### Conclusions

This study applies the tools of Network Science to provide a novel perspective on the analysis of music, offering insights into how specific structural properties of musical compositions vary across genres and change over time. Our findings open avenues for interdisciplinary research, bridging musicology, data science, and sociology to investigate how digital environments shape creativity and consumption.

Within the adopted symbolic, pitch-based framework, our analysis reveals systematic changes in network-based measures of melodic and harmonic structure over time, including increasing similarity and reduced structural differentiation in these dimensions. More broadly, our findings highlight how quantitative, reduced representations can be used to track long-term structural patterns in music. By situating these results within the broader context of technological and societal change, we provide a foundation for future research exploring the interplay between creativity, culture, and technology, potentially through the integration of complementary musical representations. At the same time, while we observe reduced structural complexity within the dimensions analyzed here, it remains possible that musical complexity has shifted toward other modes of expression not captured by the present framework. Future work combining multiple analytical approaches could help test this hypothesis and explore the evolution of musical complexity across different dimensions.

## Methods

### Dataset

#### MIDI collection

The Musical Instrumental Digital Interface, in short MIDI, is a widely used standard for representing musical information in a digital format. Rather than storing audio, the MIDI format encodes musical events such as note pitches, durations, and timing, along with performance controls such as tempo changes. Specifically, MIDI data is organized into channels, allowing multi-instrument encoding. This representation renders it particularly suitable for music information retrieval tasks.

In this study, we employ the MetaMIDI Dataset (MMD)^48^, a publicly accessible collection of more than 400,000 MIDI files. Specifically, we focus on the subset containing MIDI files that are annotated with a title, artist name, and at least one music genre. We point to the dataset’s official GitHub repository^61^ for further details about its construction.

From this initial sample of approximately 160, 000 MIDI files, we keep only tracks with genres containing at least one keyword between “rock”, “pop”, “electronic”, “classical”, “jazz”, “hiphop”, or “hip hop”. Note that a single track from this list may be associated with multiple genres. We further refine this selection by keeping only MIDIs longer than 60 seconds that can be successfully parsed by the R library tuneR^62^. This filtering procedure results in approximately 40, 000 MIDI files.

#### Spotify metadata

To enrich our dataset, we conduct a data collection phase on Spotify using the R package spotifyr^63^, a wrapper for the official Spotify API. First, we preprocess the title and artist associated with each MIDI removing:

1. everything that appears after the word feat. of ft.;
2. everything appearing between parentheses;
3. all the non-alphanumerical characters.

Then, for pieces with multiple authors ($\approx 22.15 \%$ of our dataset), we select only the first one. The rationale behind this choice is that, in most classical pieces, the original composer is listed first, followed by the names of performers. Moreover, in the main analysis, we usually do not consider artists, reducing the impact of this choice.

Using these cleaned pieces and artist names, we gather information from Spotify. In particular, for each artist in our dataset, we use the search_spotify function to retrieve their Spotify ID, name, popularity, number of followers, and associated genres. This process is repeated for every unique artist.

Next, for each piece *s* composed by artist *a*, we use the search_spotify function using the query “track: *s*, artist: *a*”, that returns a set of maximum 50 songs matching the query. To identify the correct one, we first filter for tracks where *a* is listed as one of the composers. Then, we select the piece whose name has the smallest Levenshtein distance from *s*. If multiple pieces meet this criterion, we select the one with the earliest album release date to avoid remastered or live versions. By doing so, the MIDI files are matched with their corresponding Spotify metadata, including the ID, name, popularity, and the release date of the album they belong to.

**Fig. 6:**
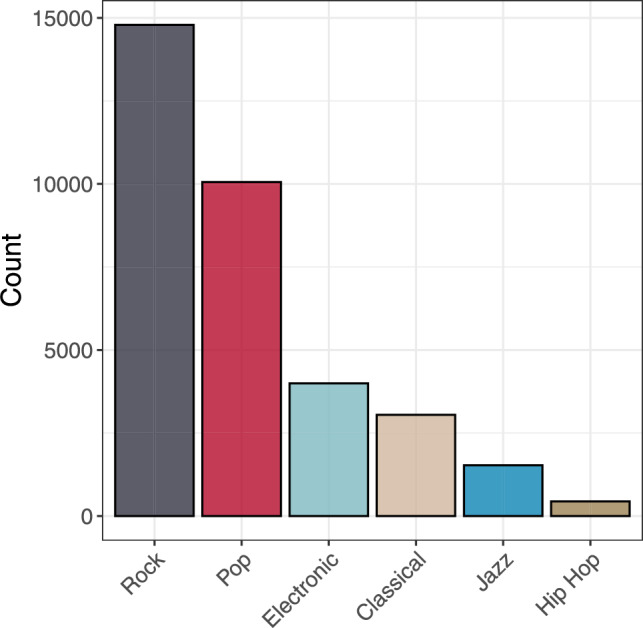
Distribution of genres in our dataset. Recall that one MIDI may be associated with multiple genres.

To ensure that only distinct pieces are considered, we select a subset of the MIDI files with unique Spotify IDs, resulting in a final dataset of 21,480 unique musical pieces. Figure 6 shows the distribution of genres in this final dataset.

### Network construction

Starting from our Dataset, we use *tuneR* library to read MIDI files as data tables containing (among others) time, note, channel, and track of each MIDI event. This allows us to construct directed networks in which nodes are distinct notes and edges indicate a transition between the two nodes, weighted by how many times it occurs. More in detail, suppose that *x*, *y* are two notes played in a certain MIDI file. A directed edge between *x* and *y* means that *y* follows *x* at least once, with the weight $w_{xy}$ counting how many times this specific transition occurs. Before proceeding, we clarify that we have decided to construct networks starting from notes for several reasons. First of all, since our analysis spans several centuries and diverse musical genres, notes represent the fundamental unit common to all of them. In contrast, the role and function of chords vary significantly across genres and centuries. For instance, in Baroque music, compositions are often built from interwoven melodic lines rather than sequences of block chords. This approach differs markedly from modern genres such as Hip-Hop or Electronic music. Moreover, in a chord-based model, much of the internal complexity of note sequences is lost—for instance, it becomes impossible to distinguish between an intricate arpeggio and a simple block chord. Such distinctions are crucial, as they reflect different compositional structures. In our note-based approach, these details are captured through note-to-note transitions over time, which more accurately describe the specific melodic and harmonic pathways explored by each composition. However, we acknowledge that other works, with more time-limited datasets, have instead employed chord-based models as well^41,64^.

In the main analysis, to focus only on transitions between different notes, we delete all loops, i.e. we set $w_{xx} = 0$ for all $x \in V$, thus obtaining simple networks. In the case of multiple notes played at the same time (e.g., chords), edges are drawn between all the notes played at first and second times, as in a complete bipartite graph. Note that we apply this procedure to each channel separately to avoid mixing up different instruments. Further, we ignore the channel associated with drums.

Figure 1 shows examples of networks gathered from different musical genres.

### Null models

We adopt two different models, one preserving the degree distribution and the other keeping the strength distribution of nodes.

In the first case, we apply a rewiring of the edges, without considering the weights, akin to the procedure described in^65^. In this way, we maintain the number of transitions that start between each note, but we change how they are connected, possibly breaking common musical patterns.

In the latter case, we use a model previously used in many works involving weighted directed graphs^66,67^. This model locally reshuffles the weights of the outgoing links, without changing the connections. Note that, from a musical point of view, this model keeps all musical choices (such as melodies or chords) intact, changing only the number of transitions between note pairs.

### Networks’ embeddings

To obtain a comprehensive picture of the properties of the MIDIs, we embed each network into a high-dimensional space using two different approaches. The first approach associates each network *G* to a $12-$dimensional vector $v_G$ in which each position is associated with a musical interval (see Table 1). In particular, $v_G[i]$ contains the number of times the interval *i* appears in the MIDI. For comparison purposes, we normalize each vector by requiring $|| v_G ||_2 = 1$. In this space, two networks are close if they share similar intervals, suggesting similarity from a musical point of view.

The second approach involves using graph2vec^68^, a well-established algorithm to create network embeddings. Specifically, we employ the implementation available in the Python package karateclub. The results using this latter approach are in SI.

We highlight that these two methods capture completely different network properties. Indeed, the former relies on purely musical features, whereas the latter exploits the topological properties of the networks.

### Network measures

Networks are widely used across various fields and numerous metrics have been developed to measure their properties. In this section, we recall some measures, focusing on their interpretation in our context.

*Density:* the density of a network is defined as its number of edges over the maximum possible i.e. $d = \frac{|E|}{|V||V-1|}$. In particular, the density measures how many note transitions have occurred compared to the possible ones. Hence, high values may suggest higher complexity, even if the measure itself does not contain any information about the type of transition.

*Reciprocity:* in a directed network, an edge $(i,j) \in E$ is *reciprocated* if also $(j,i) \in E$. However, instead of simply computing the fraction or reciprocated edges *r*, it is common^69^ to define reciprocity as

1 $$\begin{aligned} \rho = \frac{r - a}{1 - a} \end{aligned}$$

where *a* is the network density. This measure is bounded in $[-1,1]$, where positive values suggest that reciprocated links appear more than expected at random. The opposite is true when $\rho < 0$. Values close to 0 suggest a behavior comparable to a random model.

To take into account the role of edges’ weight we adopt the notation and measures introduced in^70^. In particular, the authors define the *weighted reciprocity* as

$$r = \frac{W^\leftrightarrow }{W}$$

where *W* is the sum of the weights of the network, while $W^\leftrightarrow$ is the total reciprocated weight. Without delving further into the definitions, the interested reader can find more information in^70^.

Based on the previous measures, we compare the value of *r* with those of a null model computing

$$\rho _w = \frac{r - r_{NM}}{1 - r_{NM}}$$

where $r_{NM}$ is the value obtained in a randomized network following the procedure highlighted in Section “Null models”. Note that the interpretation of the measure’s value is the same as (1).

*Mean node entropy:* entropy is a widely used measure of distribution concentration, originally defined by Shannon^71^. For a random variable $X$ with image $\mathcal {X}$, entropy is computed as:

$$H(X) = - \sum _{x \in \mathcal {X}} p(x) \log p(x),$$

where $p(x) = P(X = x)$. In the case of a distribution concentrated at a single point (i.e., a delta-like distribution), entropy reaches its minimum value, $H(X) = 0$. Conversely, a uniform distribution achieves the maximum possible entropy, $H(X) = \log (n)$, where $n = | \mathcal {X} |$. For comparative purposes, we normalize $H(X)$ to the interval $[0,1]$ by dividing it by its maximum value, $\log (n)$. We employ entropy to measure the degree of heterogeneity in the out-weight distribution of each network node.

For each node *i*, we define the transition probability of the directed edge (*i*, *j*) as $p_{ij} = \frac{w_{ij}}{\sum _j w_{ij}}$. Obviously, $\sum _j p_{ij} = 1$, thus defining a probability distribution.

Then, for each node *i*, we compute the entropy $H(X_i)$ associated with the transition probability. $H(X_i)$ is close to 1 if $p_{ij} \approx \frac{1}{k_i}$ for $j \in N(i)$, and it is close to 0 if only a links carry the majority of the weights. Finally, for a given network *G*, we compute $\bar{H} = \frac{1}{N} \sum _{i = 1}^N H(X_i)$ to measure the expected node heterogeneity.

Values close to 1 suggest that transitions among connected notes occur almost uniformly. On the other hand, lower values indicate a preference through a subset of the connected notes (i.e. higher probabilities of transition).

*Global Efficiency:* the *global efficiency*^72^ of a network *G* is a measure of how well the network can efficiently spread information in parallel, and it is defined as

2 $$\begin{aligned} E(G) = \frac{1}{|V||V-1|} \sum _{ij} \frac{1}{d_{ij}} \end{aligned}$$

where $d_{ij}$ is the length of the shortest path between *i* and *j*. Note that $E \in [0,1]$, where $E \approx 1$ indicates very well-connected (i.e. low distances) nodes.

For weighted networks, $d_{ij}$ is the shortest weighted path between *i*, *j*. Since our networks have integer weights, the measure remains bounded in [0, 1], maintaining the same interpretation.

**Fig. 7:**
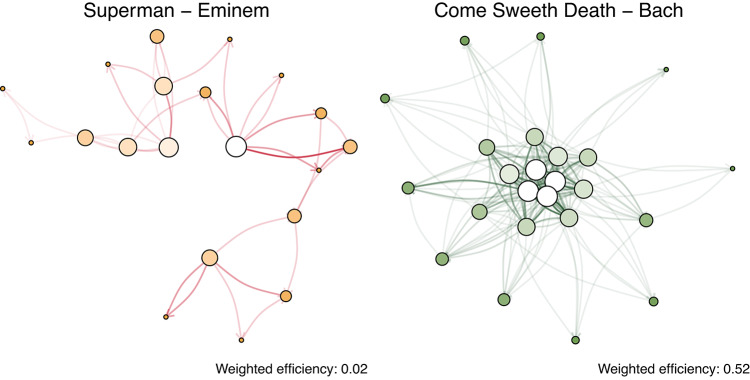
Examples of two musical networks with low (left) and high (right) weighted efficiency. Low values correspond to networks with frequent repetitions and limited melodic variation, while high values indicate greater diversity and in note transitions. This example helps understand our interpretation of complexity.

In the case of musical networks, high weighted efficiency values are obtained when the network has shorter weighted paths, suggesting pieces with non-repetitive melodies and higher musical variance. To help the reader visualize the differences highlighted by this metric, Fig. 7 illustrates the contrast between low and high values of weighted efficiency.

### Release date collection

In some cases, Spotify may report release dates that differ from the actual ones. This is especially common for remastered or reissued albums, where the reported release date corresponds to the newer version rather than the original release. Such differences may become an issue when analyzing the complexity of musical pieces or their popularity over time.

To address it, we developed a heuristic based on prompting a Large Language Model (LLM), specifically Gemini, developed by Google. In detail, we perform queries to model “gemini-1.5-flash” via the official API, which is freely accessible (https://ai.google.dev/gemini-api/), and ask the model to respond with the release date of a musical piece given its name and its artist’s name. Additionally, we ask the model not to provide a date if it is uncertain about it.

Since LLM outputs involve an inherent element of randomness and are known to contain so-called “hallucinations”, to minimize the impact of the model errors, we set a threshold on the release date of each genre. Specifically, for Rock, Pop, Electronic, and Hip Hop, we included only pieces released after 1950. For Jazz, we included pieces released after 1900. Moreover, we also delete all pieces incorrectly classified after 2021, since the dataset was released in that year.

Finally, to check the robustness of our procedure, we validate the release dates provided by Gemini and Spotify against a manually annotated sample of 100 musical pieces. For further details, Section 4.1 of the Supplementary Information provides a complete discussion of our approach. Our results, summarized in Figure S11, show that while Spotify tends to correctly associate release dates for pieces from 1980 onward, older entries are better classified by Gemini. This finding aligns with our expectations: older music is often performed more recently by contemporary musicians, leading Spotify to misclassify these pieces as new rather than older works.

For this reason, we have chosen to use Gemini’s estimates for pieces released before 1980, while retaining Spotify’s release date for more recent instances, thereby taking advantage of the strengths of both methods.

## Supplementary Information

Supplementary Information.

## Data Availability

This work uses the MetaMidi dataset, hosted on Zenodo and accessible upon request to the authors of the dataset. Instructions for accessing the dataset are available at: https://zenodo.org/records/5142664

## Code availability

All analyses were conducted using *R version 4.4.1*, a statistical computing environment available at https://www.r-project.org/. The following R packages were used: *igraph* (version 2.0.3) – Network construction and analysis. https://igraph.org/r/. *tuneR* (version 1.4.7) – MIDI data processing and note extraction. https://cran.r-project.org/package=tuneR. *dplyr* (version 1.1.4) – Data manipulation and transformation. https://cran.r-project.org/package=dplyr. *tidyr* (version 1.3.1) – Data reshaping. https://cran.r-project.org/package=tidyr. *data.table* (version 1.16.0) – Efficient data handling. https://cran.r-project.org/package=data.table. *ggraph* (version 2.2.1) – Network visualization. https://cran.r-project.org/package=ggraph. *ggplot2* (version 3.5.2) – Data visualization. https://cran.r-project.org/package=ggplot2. *tabr* (version 0.5.1) – Music theory utilities and interval computation. https://cran.r-project.org/package=tabr. The code used to construct the musical transition networks from MIDI files and to compute the interval-based embeddings is publicly available in the associated OSF repository^73^. The repository also includes a reproducible test script based on an example MIDI file of J. S. Bach’s *Prelude No. 2 in C minor* (BWV 847), demonstrating the complete workflow from MIDI input to network construction and embedding extraction.
