# Supplementary material for: Decoding the evolution of melodic and harmonic structure of Western music through the lens of network science
Source: Sci Rep. 2026 Apr 23;16:11121. doi: 10.1038/s41598-026-42872-7 (PMC13106658; doi:10.1038/s41598-026-42872-7)
Supplement: Supplementary file 1 — Supplementary Information. [file 41598_2026_42872_MOESM1_ESM.pdf]

Decoding the evolution of melodic and harmonic structure of Western music through the lens of Network Science

Supplementary Information

Contents

1 Statistical tests **2**

2 Analysis with alternative measures of complexity **2**

2.1 Entropy of Networks 2

2.2 Effective resistance 4

3 Robustness checks for time analysis **6**

4 Interval embeddings **7**

4.1 Full UMAP dimensionality reduction 7

4.2 `graph2vec` embeddings 7

4.3 Comparison with null embeddings 8

5 Time analysis **9**

5.1 Robustness of release date estimation 9

5.2 Components interpretation 10

5.3 Result of Mann-Kendall 11

1 Statistical tests

In this section, we describe the procedure applied to compare different distributions and discuss their results. In particular, to assess if there are differences in the distributions of Fig. 1(b), we employ a two-sample Mann–Whitney U test [1]. To account for multiple comparisons, we employ the standard Bonferroni-Holm correction [2].
The corrected $p-$values resulting from coupled tests are depicted in Fig. S1.

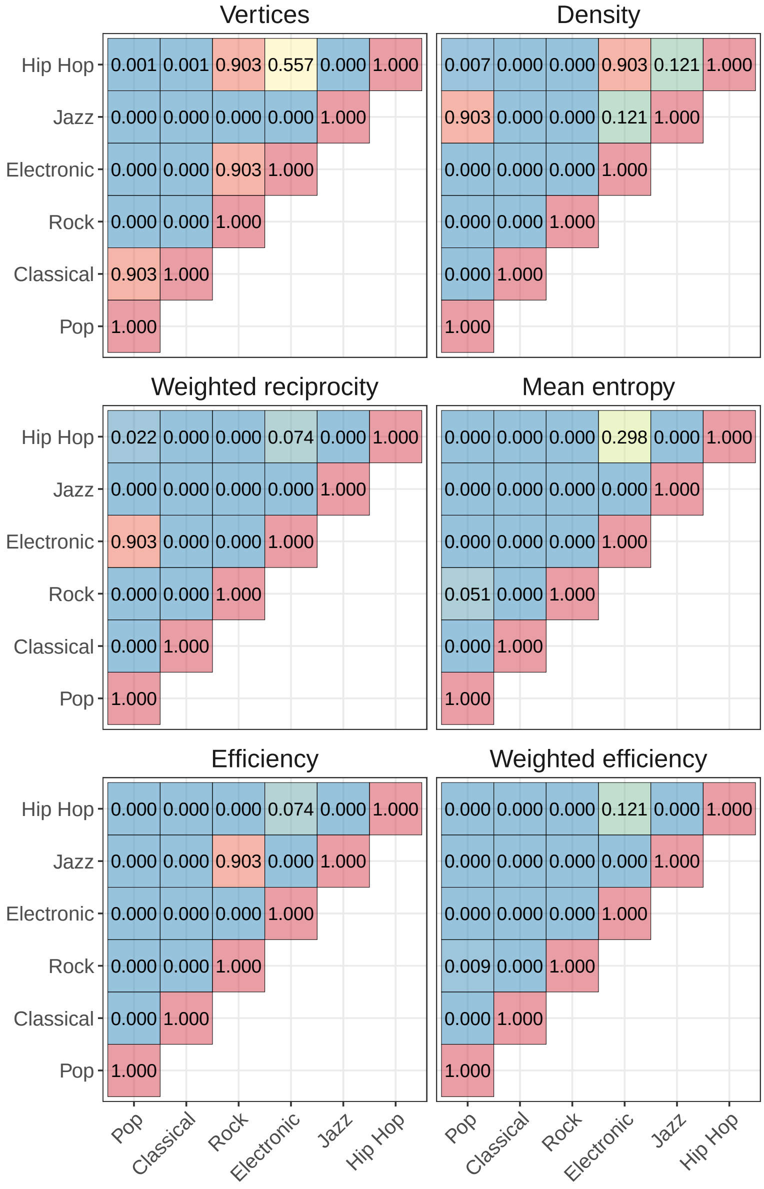

Figure S1: Corrected $p-$values of the Mann-Whitney U test for distribution coming from different genres.

Even if most tests reject the null hypothesis, there are several similarities between Rock, Pop, Electronic and Hip Hop genres. Classical music exhibits a distribution comparable with other genres only for vertex count, while Jazz music shows comparable values of Density and Efficiency with Pop and Rock music, respectively.

2 Analysis with alternative measures of complexity

2.1 Entropy of Networks

To study the spreading properties of a network and its overall information, it is common to focus on the Markov chain associated with its structure. In more detail, we can measure the information

contained in node transitions using the Shannon Entropy [3]. Formally, let us consider a weighted directed network G and denote as P its associated stochastic matrix. The entropy at the node level is:

$$H_i = -\sum_j P_{ij} \log P_{ij}. \tag{1}$$

Instead, to compute the entropy of the whole network, it is necessary to weight the contribution of each node i by the stationary distribution of each node π_i [4], that is:

$$H = \sum_i \pi_i H_i.$$

Unfortunately, for directed networks, there is no closed form for the stationary distribution π, which instead depends on the specific structure of the network [5] and may not be unique in the case of non-strong connected networks. To guarantee the existence and uniqueness of π in such cases, we add a small damping probability akin to the page rank procedure [6], i.e. we correct P considering

$$\bar{P}_{ij} = (1-\alpha)P_{ij} + \alpha\frac{1}{n},$$

using $\alpha = 0.05$. We then compute numerically π and the entropy of our collection of networks. Hence, we use these values to repeat the main analysis of the paper. The results are shown in Fig. S2.

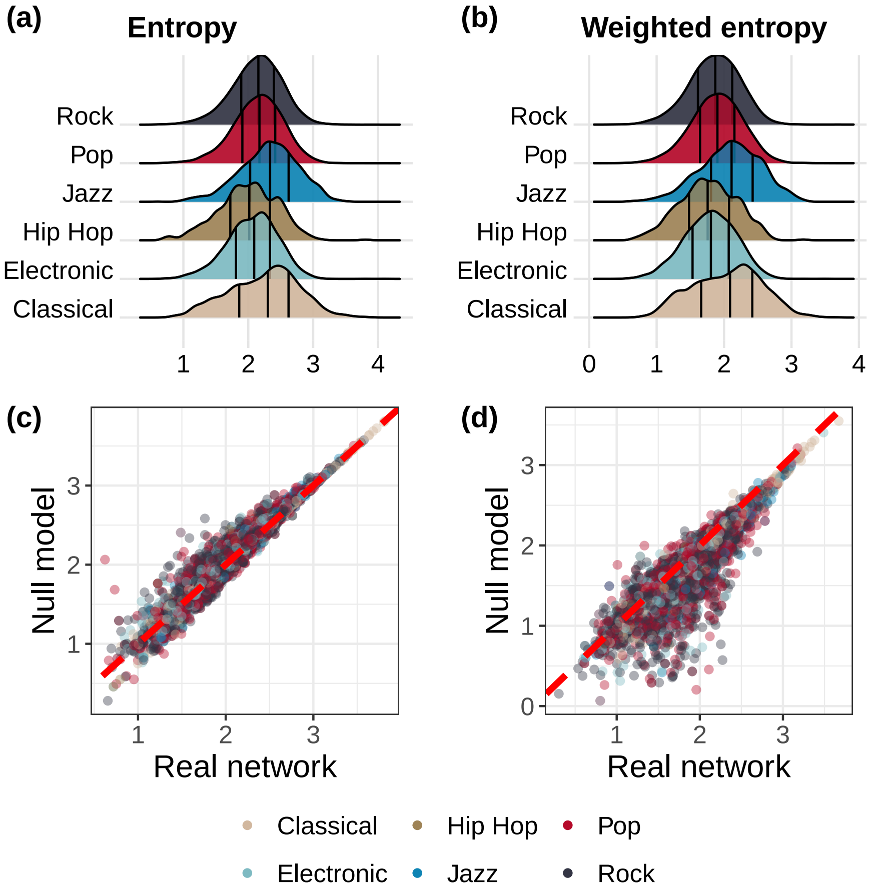

Figure S2: Distribution of (a) entropy and (b) its weighted counter part. (c), (d) shows the comparison of the values with the ones obtained from appropriately randomized versions of the networks.

The results closely resemble the ones obtained with efficiency in the main paper, since Jazz and Classical music exhibit greater information content compared to other genres. Additionally, entropy tends to have higher values in the randomized versions of the networks, although this trend reverses when weights are taken into account.
This highlights the critical role of weights in capturing the musical properties of the network.

Finally, we explore the decade-mean evolution of Entropy for each genre, akin to the procedure described in the main paper. The results of the analysis are depicted in Fig. S3.

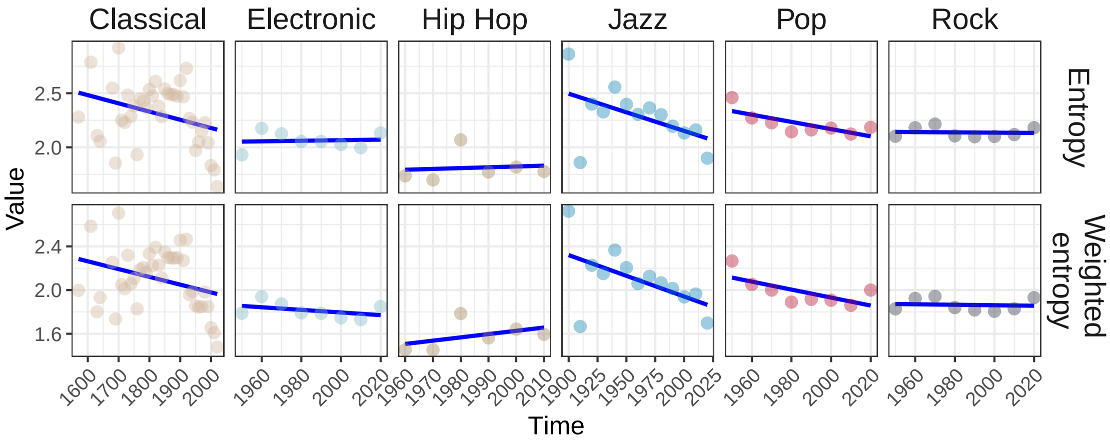

Figure S3: Trend evolution of decade-mean entropy and its weighted counterpart.

Notably, the observed trends closely resemble the ones obtained with efficiency. In particular, Classical music shows a notable decrease in Entropy in the last century, while Jazz exhibit an initial increase followed by a decrease. The other genres maintain similar constant values.

2.2 Effective resistance

To study the robustness of networks, it is common to consider, as a fundamental model, an analogy of the network in which edges represent resistors and an electrical current flows between the nodes. A fundamental question in this domain is how to quantify the overall resistance of the network, or more specifically, the resistance between any two arbitrary nodes. This measure is known as the effective resistance.

Formally, consider an undirected connected network $G = (V, E)$, where V is the set of nodes and E is the set of edges. We suppose that each edge (i, j) is associated with a positive resistance $r_{ij} > 0$, which models the resistance in that edge. In the case in which the edges are associated with weights w_{ij} representing the strength of interactions, it is common to define $r_{ij} = \frac{1}{w_{ij}}$.

The effective resistance Ω_{st} between two distinct nodes, s and t, is then defined as the potential difference V_{st} required between them to drive one unit of current $(I = 1)$ from s to t. This definition assumes that current enters the network only at node s and exits only at node t.

Mathematically, this can be expressed using Ohm's Law on the entire network:

$$\Omega_{st} = \frac{V_s - V_t}{I}$$

where I is the total current flowing from i to j, and V_i and V_j are the electrical potentials at the respective nodes. Interestingly, it is possible to compute the effective resistance using the Moore-Penrose pseudo-inverse of the Laplacian matrix of the network L^+ [7, 8]. Mathematically speaking, we obtain that:

$$\Omega_{st} = L^+_{ss} + L^+_{tt} - 2L_{st}. \tag{2}$$

Finally, the effective resistance of the network is defined as $\Omega = \sum_{s<t} R_{st}$. In the case of a weighted network, the result still holds considering the weighted Laplacian, with edge weights equal to r_{ij} (i.e. $\frac{1}{w_{ij}}$).

The concept of effective resistance, however, transcends its origins in electrical engineering. It has proven to be a powerful and robust concept in modern graph theory and network science. It is intrinsically related to the properties of random walks on graphs [9], and it is a valuable tool for measuring distance or connectivity between nodes, as it captures the global structure of the network, including the multiplicity of paths and potential bottlenecks, with applications in diverse fields [10, 11, 12].

In our context, effective resistance is a valid alternative to measure the complexity of musical networks. In fact, it is less susceptible to noise than the shortest path distance computation in the global efficiency, and it is normalized by network size. Given its properties, we argue that lower values of the metric correspond to higher musical complexity. A low resistance indicates the presence of multiple paths with easy interaction, and thus greater variability.

In this section, we present the main analyses of the study using Ω as an alternative measure of complexity. Specifically, we compute the measure on the undirected version of the networks, using as edge weights the sum of the reciprocated directed edges. Moreover, for disconnected networks, we sum the value of Ω for each connected component. These choices ensure both easier interpretation and more stable computation of the metric, which are desirable features in this context.

Figure S4 shows the resulting distribution of Ω in our network and a comparison with the values obtained using a weighted null model to randomize edges' weights, as explained in the Methods section of the main paper.

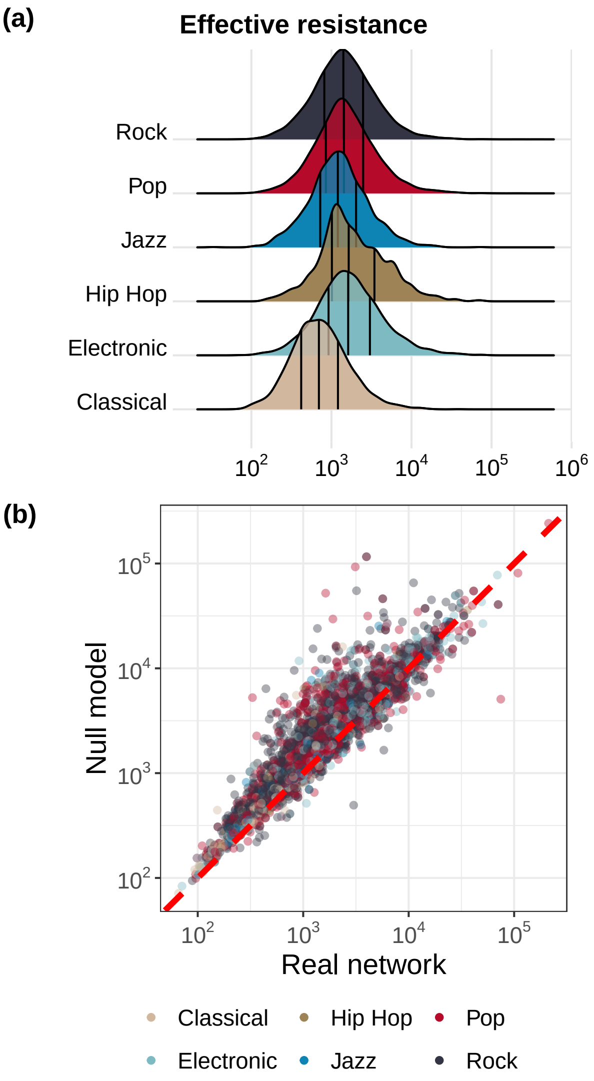

Figure S4: Distribution of (a) Ω and (b) comparison of the metric with the values obtained from appropriately randomized versions of the networks.

Consistent with the main results, we find that Classical and Jazz music exhibit lower values of Ω, indicating greater musical complexity. At the same time, we also observe that randomized networks display higher Ω values. Notably, these results are also consistent with the findings based on entropy (Figure S2).

Finally, we also explore the decade-mean evolution of Ω for each genre, as in the main paper. The results are shown in Figure S5.

Also in this case, we observe the same trends highlighted with both weighted efficiency and entropy. Classical and Jazz music exhibit a decreasing trend of complexity, with values comparable to

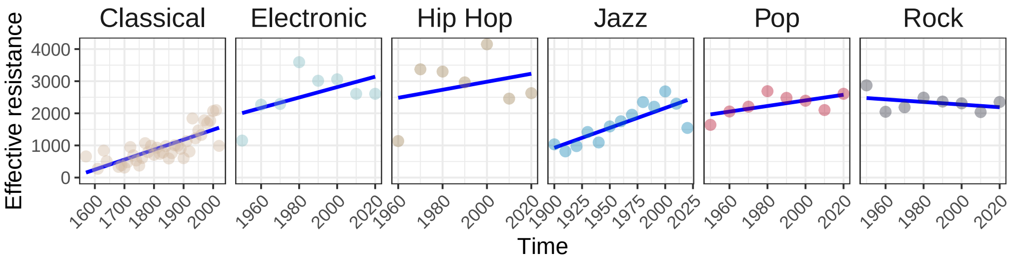

Figure S5: Trend evolution of decade-mean Ω for each musical genre.

modern genres. Notably, in this case, we also find this trend in pop music, which seems to have increasing values of Ω.

3 Robustness checks for time analysis

As discussed in the main paper, music production was much more limited in earlier periods than in modern ones. Paradoxically, this could suggest that data from older eras are actually more complete and representative than those from recent times. Consequently, there is a risk of overestimating the overall musical complexity of these earlier periods, as our dataset focuses primarily on the most popular compositions.

In this section, we perform two analyses to determine if this is really the case in our dataset.

In the first one, we selected, from each musical era (as defined in the section "Tracing musical evolution over time" of the main paper), the 100 musical pieces with the highest Spotify Popularity scores. While Spotify Popularity has an opaque definition and may change over time depending on platform listenings, we believe that, for this purpose, it is perhaps the only available metric that allows to approximate the concept of "popular music" even for earlier eras. Indeed, although this correspondence may not always hold perfectly, it is likely that the pieces that remain popular today from past eras were also popular at their time. Therefore, the goal of this analysis is to explore which patterns of musical complexity emerge when extracting a balanced sample of popular songs from each historical period.

Figure S6(a) depicts the weighted efficiency values for this balanced sample. Interestingly, we observe greater variance in musical complexity during the older eras compared to more recent ones. Before 1950, musical complexity was on average higher, but this also suggests that not all compositions from earlier periods maintained such elevated levels of complexity. In contrast, popular music from more recent decades displays both lower average complexity and lower variance, with the lowest values recorded between 1980 and 1999. Notably, from 2000 onward, although overall complexity remains relatively low, a small subset of higher complexity compositions begins to re-emerge. For clarity, Table S1 reports the standard deviation for each group, confirming these observations. Overall, the analysis based on a balanced dataset of popular songs supports our findings and indicates that the risk of overestimating the complexity of earlier periods is limited.

However, given the limitations of the popularity metric, we decided to conduct further investigations. Specifically, we repeatedly extracted samples of 100 musical pieces (for 500 iterations) from the two eras preceding 1950 (namely, "<1900" and "1900–1949"), each time computing the mean value of the resulting distribution. With this process, we aim to understand what happens if we consider less complete samples for the older eras for estimating complexity.

Figure S6(b) shows the distribution of the mean values obtained with this pipeline. The results show that, even after downsampling, the older periods still exhibit higher mean values than the more recent ones—specifically, 0.224 for "1950–1979," 0.196 for "1980–1999," and 0.186 for ">2000", thus limiting again the risk of overestimating complexity.

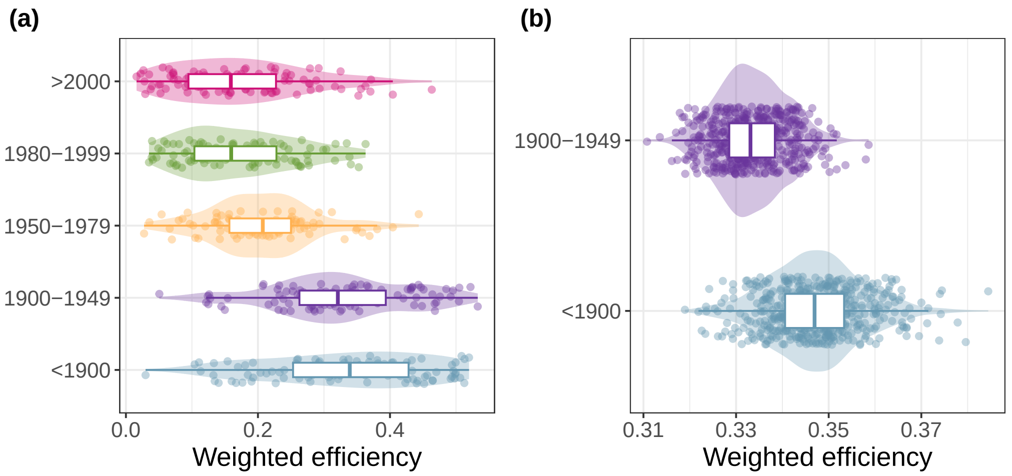

Figure S6: (a) Weighted efficiency values for the 100 songs with the highest Spotify popularity. (b)

	Musical eras	Standard deviation
1	<1900	0.119
2	1900-1949	0.103
3	1950-1979	0.079
4	1980-1999	0.083
5	>2000	0.097

Table S1: Standard deviation obtained from the samples of 100 most popular songs in each musical era.

4 Interval embeddings

4.1 Full UMAP dimensionality reduction

Figure S7 shows the 2−dimensional coordinates of interval embeddings obtained using UMAP. Interestingly, a distinct cluster of Classical music emerges in the upper-left portion of the plot, while Jazz songs form a noticeable cluster in the mid-upper region. In contrast, the remaining genres exhibit more mixed behavior and lack clear clustering.
The relatively high values achieved by artists indicate limited exploration. However, this may again reflect a universal preference for specific musical patterns, as certain transitions may be generally considered dissonant.

4.2 `graph2vec` embeddings

We replicate the main analysis of the paper using embeddings generated by `graph2vec`, an algorithm that extends NLP techniques to learn networks' features and embed them into a suitable high-dimensional space.
Figure S8(a) shows the center of mass of each genre according to the 128−dimensional embeddings created by the algorithm.
Notably, we observe a small distance between Classical and Jazz music. Further, in this case, Hip Hop tends to cluster away from the other genres.
To interpret the role of components, panel (b) shows the correlation between measures and coordinates. In this case, the first component can be interpreted as a mixture of topological and weighted properties of the network, while the second is more hardly interpretable. Notably, the high values of Classical and Jazz on the first component confirm again their higher complexity.
Finally, we observe a negative correlation between the GS−score and the artists' popularity ($r = -0.12, p < 0.001$) as in the main paper.
Shifting to the time analysis, Fig. S9 depicts the center of mass of each musical period, as reported in the main paper.
In this case as well, we observe a shift between music composed before 1950 and more recent pieces, which show greater similarity.

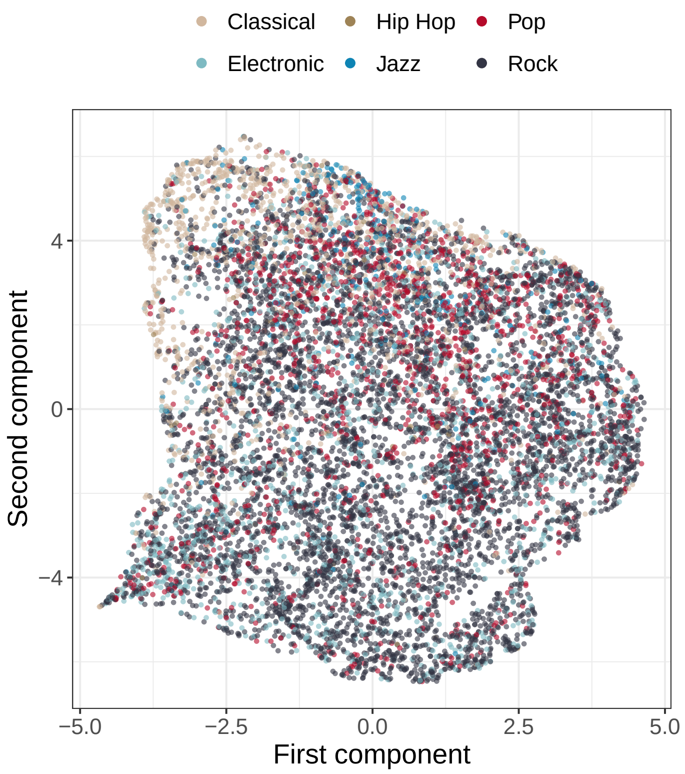

Figure S7: Low-dimensional representation of each network, with coordinates obtained from UMAP. For visualization purposes, a sample of 10^4 points is shown.

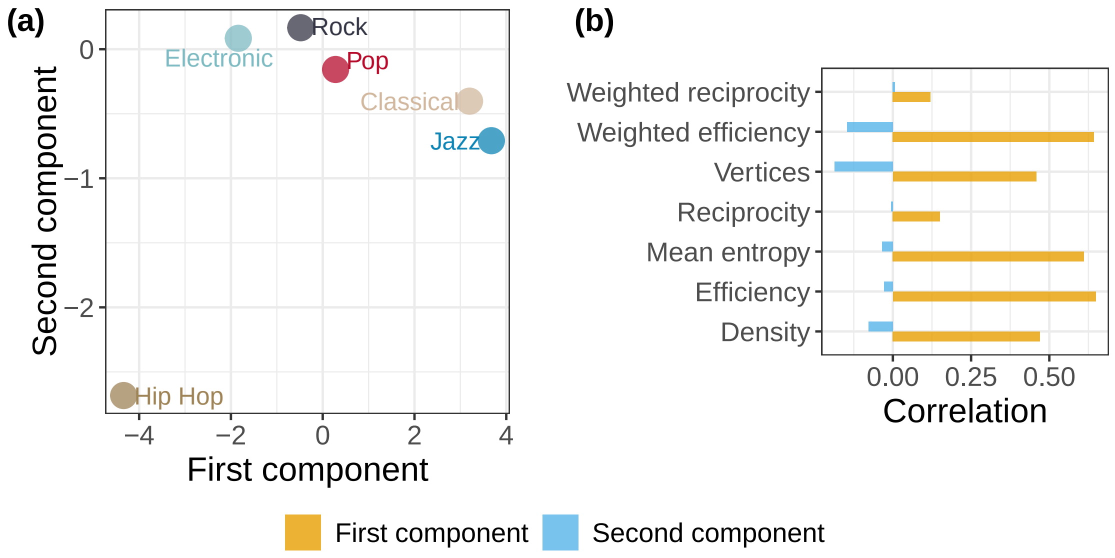

Figure S8: (a) Center of mass for each genre, computed using UMAP 2−dimensional coordinates. (b) Correlation between network measures and UMAP coordinates.

4.3 Comparison with null embeddings

To check the robustness of the interval embeddings in separating music from noise, we select a random sample of 1000 networks having at least 30 nodes and we randomize them by rewiring edges and randomly assigning weights. We then construct again the embeddings, thus obtaining a set $\mathbf{v}_{null}$ of vectors associated with randomized networks. These randomized networks serve as representations of noise, lacking any meaningful musical structure.
Then, we apply UMAP to the matrix containing these embeddings and their real counterpart. Their 2−dimensional representation is depicted in Figure S10.
Interestingly, we can observe two distinct clusters with a lower number of intersections. This cor-

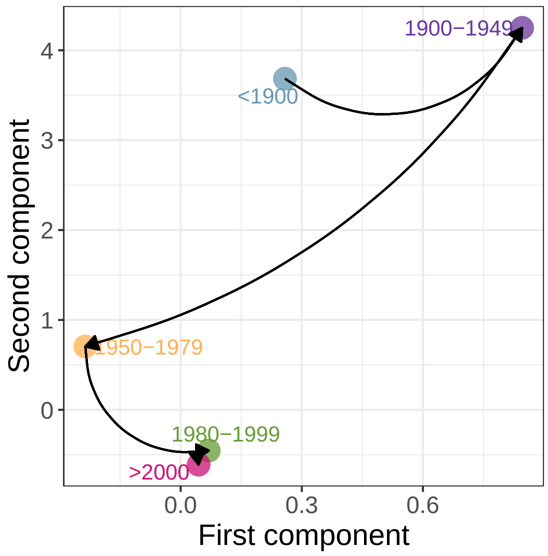

Figure S9: Center of mass of each musical period, obtained using UMAP on embeddings created starting from `graph2vec`.

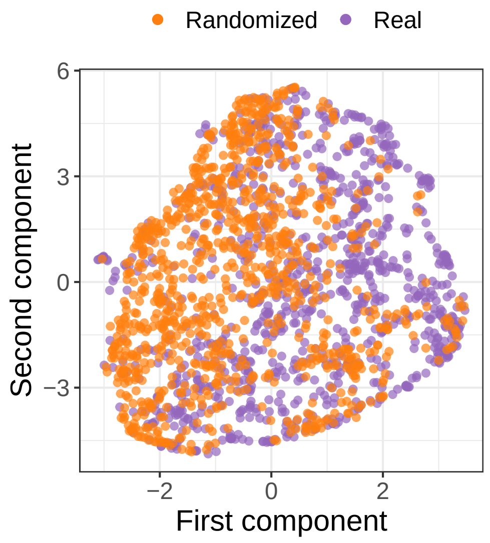

Figure S10: Results of UMAP applied a set of embeddings and their counterpart obtained after randomization of networks.

roborates the efficacy of our embeddings in capturing relevant musical properties of the networks.

5 Time analysis

5.1 Robustness of release date estimation

As detailed in the main paper, we use Gemini to estimate the release date for each song in our dataset, as Spotify's release date information is often inaccurate.
Although innovative, LLM may assign wrong release dates. However, without a ground truth, it is not easy to estimate the validity of our approach.
To try to fill this gap, we select a random sample of 100 MIDI files and manually annotate their release date. Then, we compare the original release date with that of Spotify and Gemini.
In particular, as in the main paper, we associate each song to one between 5 musical periods, namely $< 1900, 1900 - 1949, 1950 - 1979, 1980 - 1999, > 2000$ according to its release date.
Figure S11 presents the contingency matrices comparing the results of (a) Gemini and (b) Spotify in predicting the real release period of a song in our sample.

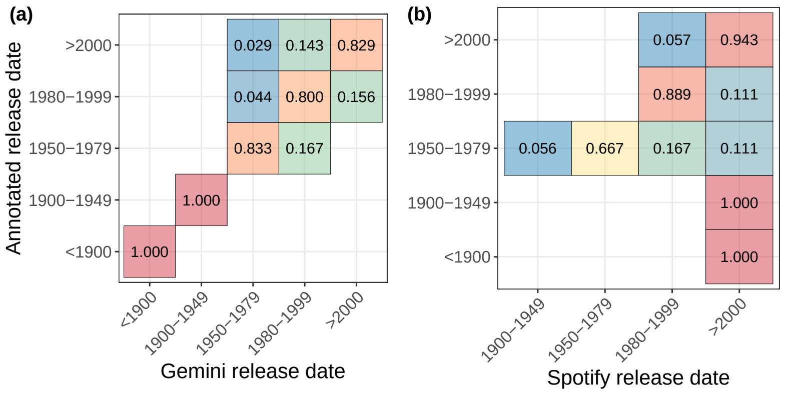

Figure S11: Contingency matrix illustrating the distribution of songs from a given musical era as classified by (*a*) Gemini and (*b*) Spotify.

While Spotify tends to correctly associate release dates of recent songs, i.e. from 1980 onward, older songs are better predicted by Gemini, which significantly outperforms Spotify in this regard. Notably, Spotify reports no songs released before 1900.
For these reasons, we have opted to use Spotify's release date for songs released after 1980, and Gemini's release date for those before, leveraging the strengths of both approaches.

5.2 Components interpretation

Akin to the procedure described in the main paper, Figure S12 shows the correlation between the two components obtained using UMAP and the (*a*) network measure or (*b*) intervals components.

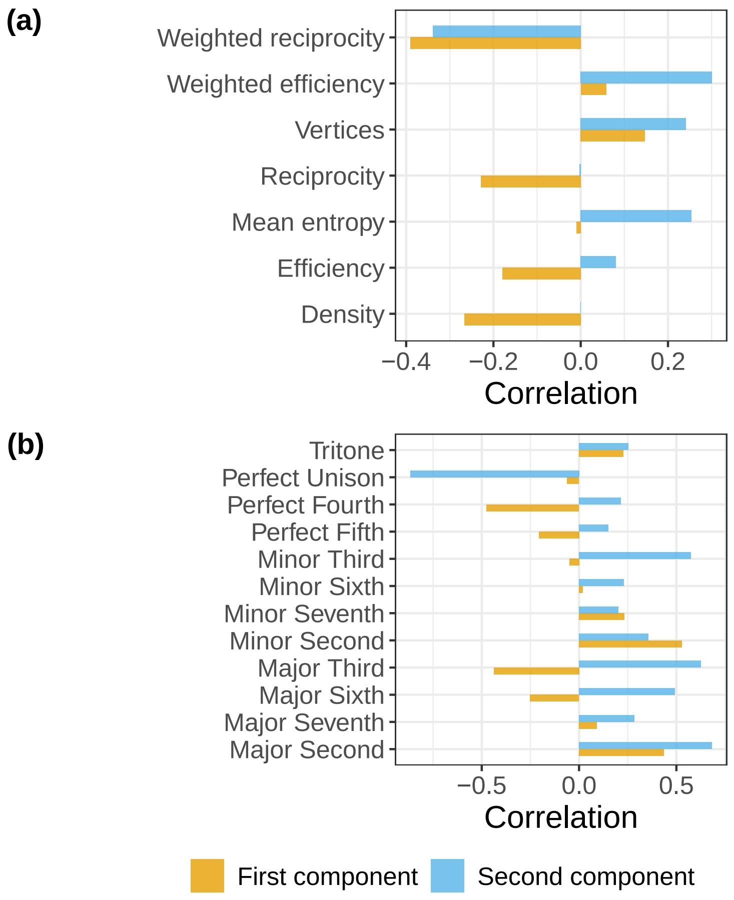

Figure S12: (*a*) correlation between measures and components. (*b*) correlation between intervals components and coordinates.

5.3 Result of Mann-Kendall

In this section we report the results of the Mann-Kendall test applied to the time-trends depicted in the main paper. The results are summarized in Table S2.

Genre	Measure	τ	p_{adj}
Classical	Efficiency	**-0.439**	< 0.001
Classical	Weighted efficiency	**-0.304**	0.007
Electronic	Efficiency	0.071	0.902
Electronic	Weighted efficiency	-0.500	0.216
Hip Hop	Efficiency	-0.143	0.764
Hip Hop	Weighted efficiency	-0.333	0.735
Jazz	Efficiency	**-0.718**	0.002
Jazz	Weighted efficiency	**-0.564**	0.009
Pop	Efficiency	0.143	0.711
Pop	Weighted efficiency	**-0.714**	0.037
Rock	Efficiency	0.643	0.071
Rock	Weighted efficiency	-0.357	0.266

Table S2: Result of Mann-Kendall tests applied to the trends of efficiency evolutions. The $p-$values have been corrected using the Holm procedure. We have highlighted the statistically significant values at the 0.05 level.

References

[1] Henry B Mann and Donald R Whitney. On a test of whether one of two random variables is stochastically larger than the other. *The annals of mathematical statistics*, pages 50–60, 1947.

[2] Sture Holm. A simple sequentially rejective multiple test procedure. *Scandinavian journal of statistics*, pages 65–70, 1979.

[3] Claude Elwood Shannon. A mathematical theory of communication. *The Bell system technical journal*, 27(3):379–423, 1948.

[4] Sean P Meyn and Richard L Tweedie. *Markov chains and stochastic stability*. Springer Science & Business Media, 2012.

[5] Richard Serfozo. *Basics of applied stochastic processes*. Springer Science & Business Media, 2009.

[6] Sergey Brin and Lawrence Page. The anatomy of a large-scale hypertextual web search engine. *Computer networks and ISDN systems*, 30(1-7):107–117, 1998.

[7] D. J. Klein and M. Randić. Resistance distance. *Journal of Mathematical Chemistry*, 12(1):81–95, December 1993.

[8] Sundaram Seshu and Myril Baird Reed. Linear graphs and electrical networks. *(No Title)*, 1961.

[9] A. K. Chandra, P. Raghavan, W. L. Ruzzo, and R. Smolensky. The electrical resistance of a graph captures its commute and cover times. In *Proceedings of the twenty-first annual ACM symposium on Theory of computing - STOC '89*, STOC '89, page 574–586. ACM Press, 1989.

[10] Arpita Ghosh, Stephen Boyd, and Amin Saberi. Minimizing effective resistance of a graph. *SIAM Review*, 50(1):37–66, January 2008.

[11] Prasad Tetali. Random walks and the effective resistance of networks. *Journal of Theoretical Probability*, 4(1):101–109, January 1991.

[12] Xiangrong Wang, Evangelos Pournaras, Robert E. Kooij, and Piet Van Mieghem. Improving robustness of complex networks via the effective graph resistance. *The European Physical Journal B*, 87(9), September 2014.
